# A Temperature Compensation Approach for Micro-Electro-Mechanical Systems Accelerometer Based on Gated Recurrent Unit–Attention and Robust Local Mean Decomposition–Sample Entropy–Time-Frequency Peak Filtering

**DOI:** 10.3390/mi15040483

**Published:** 2024-03-30

**Authors:** Rubiao Cui, Jingzehua Xu, Botao Huang, Huakun Xu, Miao Peng, Jingwen Yang, Jintao Zhang, Yikuan Gu, Daoyi Chen, Haoran Li, Huiliang Cao

**Affiliations:** 1 Tsinghua Shenzhen International Graduate School, Tsinghua University, Shenzhen 518055, China; crb21@mails.tsinghua.edu.cn (R.C.); xjzh23@mails.tsinghua.edu.cn (J.X.); chen.daoyi@sz.tsinghua.edu.cn (D.C.); 2 College of Information Science and Electronic Engineering, Zhejiang University, Hangzhou 310027, China; bthuang20@163.com; 3 Ocean College, Zhejiang University, Zhoushan 316021, China; huakunxu98@163.com (H.X.); miaopeng_xr@163.com (M.P.); 4 College of Computer Science and Technology, Zhejiang University, Hangzhou 310027, China; 13858300287@163.com; 5 Key Laboratory of Instrumentation Science & Dynamic Measurement, Ministry of Education, North University of China, Taiyuan 030051, China

**Keywords:** MEMS accelerometer, attention, temperature drift compensation, RLMD, GRU

## Abstract

MEMS accelerometers are significantly impacted by temperature and noise, leading to a considerable compromise in their accuracy. In response to this challenge, we propose a parallel denoising and temperature compensation fusion algorithm for MEMS accelerometers based on RLMD-SE-TFPF and GRU-attention. Firstly, we utilize robust local mean decomposition (RLMD) to decompose the output signal of the accelerometer into a series of product function (PF) signals and a residual signal. Secondly, we employ sample entropy (SE) to classify the decomposed signals, categorizing them into noise segments, mixed segments, and temperature drift segments. Next, we utilize the time-frequency peak filtering (TFPF) algorithm with varying window lengths to separately denoise the noise and mixed signal segments, enabling subsequent signal reconstruction and training. Considering the strong inertia of the temperature signal, we innovatively introduce the accelerometer’s output time series as the model input when training the temperature compensation model. We incorporate gated recurrent unit (GRU) and attention modules, proposing a novel GRU-MLP-attention model (GMAN) architecture. Simulation experiments demonstrate the effectiveness of our proposed fusion algorithm. After processing the accelerometer output signal through the RLMD-SE-TFPF denoising algorithm and the GMAN temperature drift compensation model, the acceleration random walk is reduced by 96.11%, with values of 0.23032 g/h$/\sqrt{\mathrm{Hz}}$ for the original accelerometer output signal and 0.00895695 g/h$/\sqrt{\mathrm{Hz}}$ for the processed signal.

## 1. Introduction

Micro-electro-mechanical systems (MEMS) accelerometers present several advantages, including compact dimensions, minimal power requirements, a lightweight configuration, and seamless integration with on-chip signal processing components. These benefits contribute to their widespread adoption, reaching consumer-oriented applications and resulting in an annual sales volume surpassing 100 million MEMS units accelerometers [1,2]. Serving as exemplary representatives of MEMS inertial sensors, gyrocopes, accelerometers, and similar devices have found extensive applications in high-precision measurement and control fields, such as consumer electronics and aerospace. This is attributed to their low cost, low power consumption, high efficiency, and high sensitivity [3].

However, inherent defects in manufacturing processes, hardware design, and the sensors themselves contribute to the significant deviation between the output signals of accelerometers and their ideal signals [4,5]. This deviation substantially diminishes the measurement accuracy of accelerometers. Moreover, with the rapid development in various industries in recent years, the requirements of the accuracy of these measurement sensors have become increasingly stringent, necessitating improvements in the accuracy of MEMS inertial devices. Currently, the main sources of errors include noise within and outside the system, as well as output drift caused by temperature changes. In recent years, numerous scholars, both domestically and internationally, have proposed various solutions based on hardware structures and subsequent software processing [6]. Significant progress has been achieved in the areas of noise reduction and temperature compensation for MEMS devices [5,7,8,9,10,11,12].

Hardware compensation primarily focuses on optimizing the structure and measurement configuration of MEMS accelerometers. Yang et al. proposed an architecture that employs on-chip temperature sensors for on-chip temperature compensation and utilizes microheaters for the on-chip temperature control of a sensor [13]. However, hardware compensation is characterized by a lengthy development cycle, high consumption, and implementation challenges. Another approach involves software compensation, achieved by establishing a compensation model to ascertain the relationship between temperature input and accelerometer output. This method eliminates the need to consider hardware constraints and, with the availability of sufficient real experimental data, proves to be more efficient in compensating effects, making it worthy of attention. Classified according to the processing method, software compensation can be divided into serial processing and parallel processing. In serial processing, the entire output signal is first denoised, followed by the establishment of a compensation model to eliminate temperature errors. However, this method of denoising before compensation may result in the loss of useful information in the original signal, thereby affecting the learning accuracy of subsequent compensation models, leading to suboptimal compensation results. Unlike the serial processing model, the parallel processing model simultaneously extracts the noise and drift components of the signal, then processes them separately for denoising and compensation, and finally reconstructs the ultimate signal. This is also the approach adopted in this study.

MEMS accelerometers may be influenced by various environmental factors in different conditions, including temperature, pressure, electromagnetic interference, and humidity. However, the main source of error in MEMS accelerometers is temperature-induced temperature drift [14,15,16,17,18]. Designing a temperature compensation model poses two fundamental challenges: addressing the high-frequency noise in components and mitigating the low-frequency temperature drift. A prevalent approach to tackle both issues simultaneously involves the parallel application of signal decomposition algorithms and temperature drift prediction algorithms to achieve temperature compensation. Among the various decomposition algorithms, Fourier transform, Kalman filtering, and empirical mode decomposition (EMD) are notable choices [19]. However, each of these methods exhibits certain limitations. Fourier transform is well suited for frequency-domain analysis of steady-state signals but proves challenging for non-steady-state or nonlinear signals, lacking temporal information. Kalman filters demand high accuracy in system models, and deviations from accurate modeling can significantly impact filtering effectiveness. As for EMD, its performance is sensitive to local extremities in the signal, leading to potential oscillations or pseudo-decomposition issues in specific signal scenarios [20,21]. In summary, Fourier transform is applicable for the frequency-domain analysis of steady-state signals, Kalman filters are suitable for linear system state estimation, and EMD is adept at adaptive decomposition of nonlinear and nonstationary signals, making it more suitable for the temperature compensation model proposed in this paper.

To address the limitations of EMD when influenced by local extremities, Smith et al. proposed the local mean decomposition (LMD) algorithm [22]. Compared to EMD, LMD exhibits greater stability, offering improved handling of nonlinear and nonstationary signals while reducing sensitivity to local extremities. The introduced parameter control mechanism enhances flexibility in the decomposition process and demonstrates excellent noise-handling capabilities, facilitating the extraction of essential signal components. Nevertheless, the LMD algorithm is not immune to issues like mode mixing and endpoint effects. In response to these challenges, Liu et al. proposed the robust local mean decomposition (RLMD) algorithm by adjusting appropriate parameters for boundary conditions, applying envelope estimation, and sifting stop criteria. The temperature compensation model presented in this paper adopts the RLMD algorithm to overcome these challenges [23]. Moreover, sample entropy (SE) is a nonlinear, model-independent metric used to characterize the irregularity and uncertainty of a signal. Its sensitivity to both randomness and periodicity in signals makes it well suited for analyzing complex, nonlinear systems. In recent years, many scholars have integrated such sequence entropy algorithms with other compensation methods to analyze nonstationary signals [24,25,26]. This reflects the growing trend of leveraging their capabilities in signal processing applications. Wei et al. proposed a variational modal decomposition optimized using a genetic particle swarm optimization variational modal decomposition parallel processing algorithm, which effectively reduces gyroscope errors associated with temperature [27].

In addition, addressing the temperature drift component requires a specific temperature drift model. Currently, deep learning is most suitable for modeling such components that lack a specific physical model but involve ample experimental data. Wang et al. proposed enhanced particle swarm optimization and support vector machine algorithms to improve the precision of fiber optic gyroscopes [28]. However, this model overlooks the characteristics of temperature, as its output is not only related to the current time but is also heavily influenced by past moments of temperature. Therefore, this paper introduces a specialized GRU-MLP-attention model (GMAN) model, incorporating gated recurrent unit (GRU), multilayer perceptron (MLP), and attention modules to handle time series information [29,30]. The model takes the past information of temperature from the previous n moments as input and predicts the temperature drift at the nth moment as output.

In this article, we propose a parallel denoising and temperature compensation fusion algorithm for MEMS accelerometers based on RLMD-SE-TFPF and the GMAN model. First, we applied RLMD to break down the accelerometer output into various product functions (PFs). Then, using SE, we sorted these PF components into noise, mixed, and temperature drift segments. Noise segments were cleared with a larger window time-frequency peak filtering (TFPF), while mixed segments were filtered with a carefully selected parameter. Temperature drift segments, being smooth curves, were left untreated. After that, we fed the processed temperature data into the GMAN model to compensate for temperature effects in the denoised signal. Finally, by putting together each segment, we reconstructed the ultimate accelerometer signal. More detailed information on the theory, experiments, and comparisons can be found in the following sections.

The contributions of this paper can be summarized as follows:

- We propose a novel GMAN model and RLMD-SE-TFPF fusion algorithm for temperature compensation and denoising of accelerometer signals. This involves modules such as RLMD, SE, TFPF, GRU, attention, etc. To the best of our knowledge, in the field of temperature compensation for MEMS accelerometers, this is the first time that time series information has been utilized for temperature drift prediction, and experimental results confirm the superiority of the proposed solution.
- Our complete temperature compensation model works in parallel. It separates the noise and drift components of the signal and then simultaneously denoises and compensates. The original signal does not experience extra loss, and there is no delay in the temperature compensation process.
- The effectiveness of our approach was validated using the Allan algorithm. The acceleration random walk (representing noise characteristics) was reduced by 96.11%, with values of 0.23032 g/h$/\sqrt{\mathrm{Hz}}$ for the original accelerometer output signal and 0.00895695 g/h$/\sqrt{\mathrm{Hz}}$ for the processed signal.

The specific content in this paper is arranged as follows: Section 2 provides a detailed introduction to the hardware structure of accelerometer and some specific parameters. Section 3 describes various algorithms mentioned in this paper and the composition of the entire temperature compensation model. Section 4 presents the experimental results and a comparative analysis of different denoising algorithms. Section 5 provides conclusions.

## 2. Structure of MEMS Accelerometer

The experimental signals collected in this article were from a self-developed single-chip capacitive accelerometer in a laboratory. Capacitive accelerometers have extensive practical applications, particularly in high-precision fields. They can ensure good device stability, have an ample measurement range, and have minimal coupling effects between orthogonal axes. Additionally, they have relatively lenient process requirements, making them ideal for optimal overall performance.

**Figure 1:**
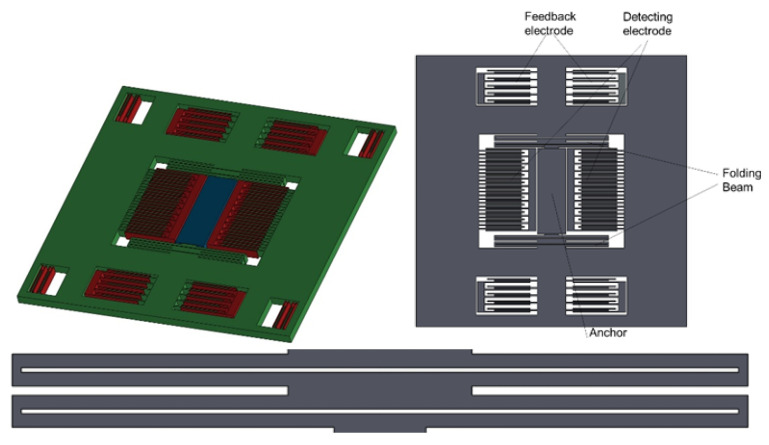
MEMS accelerometer structure.

The accelerometer’s structure is compact, with the anchor point fixed at the center to effectively increase structural efficiency and reduce the impact of thermo-mechanical stress on sensitive structures. During operation, the structure operates in a closed-loop fashion, employing uniformly spaced teeth in a fully differential comb-tooth capacitance detection and electrostatic force feedback mode. The maximum closed-loop range can reach 15 g, where g represents the acceleration due to gravity, commonly used as a unit of acceleration. The structure adopts a cruciform (cross-shaped) beam design, combining the advantages of serpentine and folded beams. It possesses moderate stiffness and the ability to release residual stress. The cruciform design contributes to a more compact overall structure of the accelerometer. The design of calibration teeth and feedback teeth ensure the stable operation of the structure in a closed-loop state. The structure of the accelerometer is illustrated in Figure 1.

This accelerometer utilizes a differential capacitive sensing circuit and closed-loop electrostatic force feedback to detect acceleration. In ideal conditions, when the acceleration input is zero, the movable mass plate is positioned at the center between the two fixed electrode plates, in a null position. With a sufficiently high loop gain and the effect of negative feedback in the closed-loop system, the electrostatic force on the side where the gap increases (due to acceleration input not being zero) causes the movable mass plate to move, generating a corresponding directional displacement.

**Figure 2:**
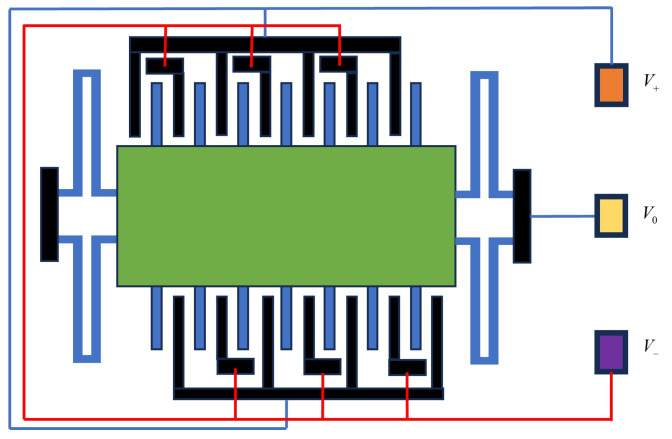
The differential comb-tooth capacitance detection model.

In the closed-loop system, if the gap on one side increases, the electrostatic force on that side also increases, and if the gap decreases, the electrostatic force on that side decreases. When the system parameters are appropriately adjusted, the overall positive feedback action during open-loop operation is prevented, ensuring that the movable mass plate remains near the null position. The differential comb-tooth capacitance detection model of this accelerometer is illustrated in Figure 2.

**Figure 3:**
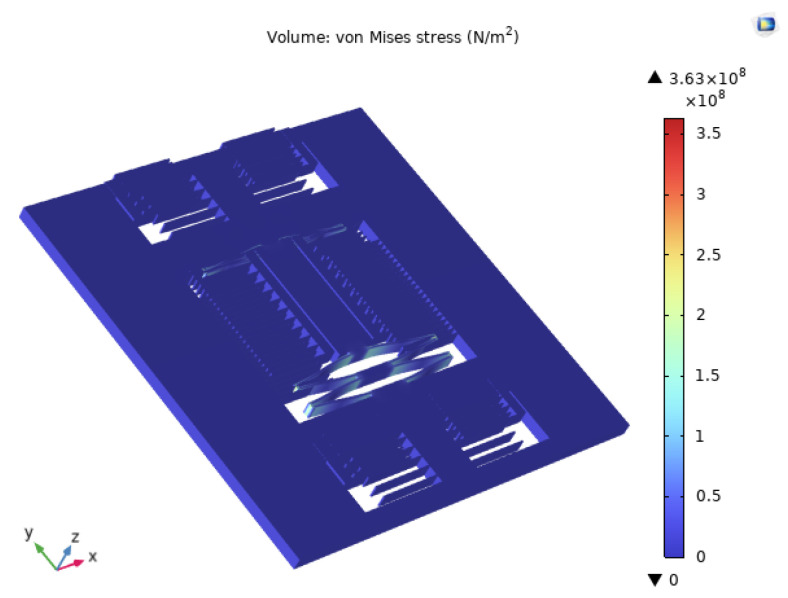
The structural stress diagrams in the horizontal and vertical axes under a 2000 g 11 ms impact.

In the design of the accelerometer’s sensitive structure, repeated shock experiments during testing can lead to fatigue damage to the chip structure, reduce the fracture strength of silicon materials, and potentially result in sensor failure. Based on finite element analysis (FEA), the structural stress under 2000 g 11 ms impact in both horizontal and vertical axes is illustrated in Figure 3. Considering the performance and manufacturing methods of accelerometers the optimized structural parameters of the sensor are presented in Table 1.

**Figure 4:**
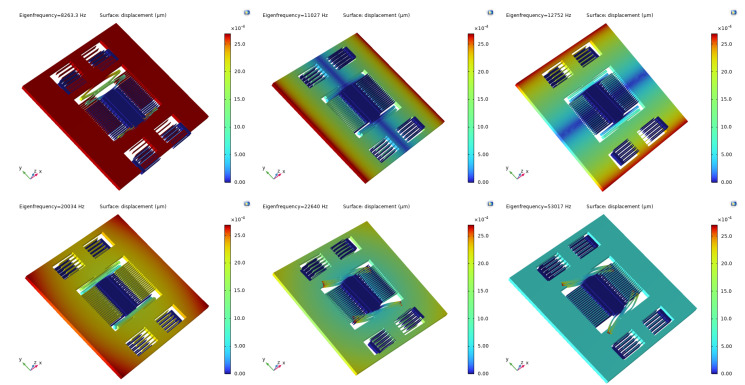
The first six modes of the accelerometer.

Modal analysis was employed to acquire the vibration characteristics of the sensor, including natural frequencies, mode shapes, and vibration stability. Figure 4 illustrates the first six mode shapes of the accelerometer, while Table 2 provides the modal frequencies. As shown in Figure 4, the first mode shape is utilized for detecting horizontal axis acceleration, with a corresponding natural frequency of 8595.3 Hz, providing the accelerometer with a wide bandwidth. This acquisition speed ensures that the accelerometer can accurately capture rapid changes in acceleration across a broad frequency range, making it suitable for a wide range of applications requiring high-frequency data acquisition and analysis. The remaining modes are considered disturbance modes, and the significant differences between operational and disturbance modes effectively mitigate coupled vibrations, enhancing the stability of the sensor’s structure.

**Figure 5:**
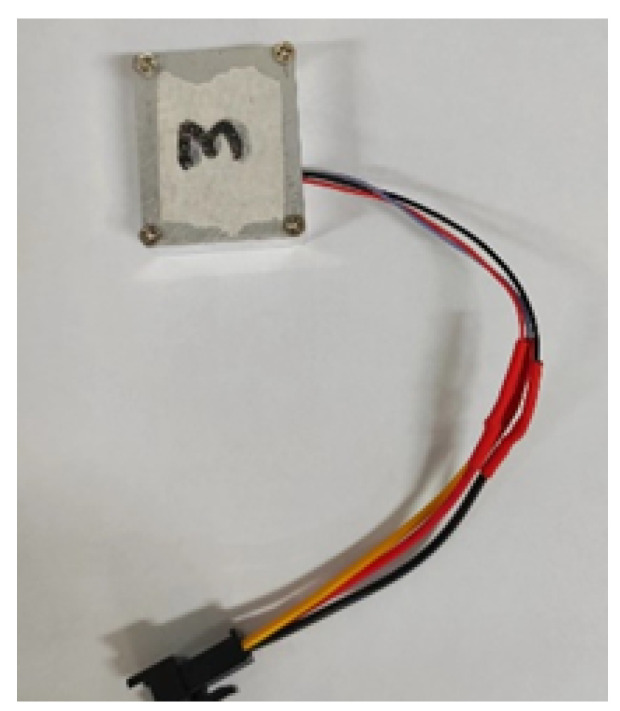
The overall packaging of the accelerometer.

The prototype of the accelerometer based on silicon fabrication has a closed-loop range of 15 g along the horizontal axis, with an axial sensitivity of 65 mV/g. The natural frequency is 8595.3 Hz. The quality factor of the accelerometer under nonvacuum atmospheric pressure is 8.54, and it demonstrates an overload capability of 2000 g, as verified using Hopkinson’s bar. Additionally, the overall appearance of the accelerometer is depicted in Figure 5.

## 3. Algorithms

### 3.1. Local Mean Decomposition

The RLMD method is grounded in the initial local mean decomposition algorithm, optimizing the latter through three criteria: filtering termination criteria, envelope estimation, and boundary conditions. This approach surpasses the LMD method in signal processing, particularly excelling in signal time-frequency analysis and feature extraction. Local mean decomposition (LMD) is an iterative signal processing technique designed to extract a set of best-fit product functions (PFs) for pure FM signals and envelope signals. The algorithm of LMD comprises a nested loop structure, with its outer loop aimed at extracting PsF from the original discrete signal, while the inner loop is dedicated to extracting pure FM and envelope signals from the PFs. The LMD process is described below, providing the fundamental principles for the proposed approach.

Step 1 Calculate the mean of the maximum and minimum points of each half-wave oscillation of the signal. For each pair of consecutive extrema $n_{i}$ and $n_{i+1}$, the ith mean value $m_{i}$ is expressed as follows:

(1) $$m_{i}=\frac{n_{i}+n_{i+1}}{2}.$$

where $n_{i}$ is the ith extrema of the signal.

Step 2 These local mean points are connected to form a smooth curve, denoted as *m*(*t*), and the corresponding envelope is obtained accordingly. The amplitude calculation for half-wave oscillations is as follows:

(2) $$a_{i}=\frac{|n_{i}+n_{i+1}|}{2}.$$

Step 3 Iterative process. The original data are subtracted from the mean curve *m*(*t*), and the resulting signal is divided by the envelope estimate *h*(*t*) to generate a pure frequency-modulated signal.

(3) $$h_{11}\left(t\right)=x\left(t\right)-m_{11}\left(t\right),$$

(4) $$s_{11}\left(t\right)=\frac{h_{11}\left(t\right)}{a_{11}\left(t\right)}.$$

where *x*(*t*) is the original data.

When $s_{11}\left(t\right)$ lacks a stationary envelope, we need to iterate this operation continuously. The number of iterations is denoted by subscript *q*. Afterward, the final envelope signal is multiplied by the frequency-modulated signal to obtain the first product function. Subtracting this product function from the original signal, the same operation is applied to the remaining signal. Following this procedure, a series of product functions can be obtained.

(5) $$\begin{array}{c} \left\{\begin{array}{cc} h_{11}\left(t\right) & =x\left(t\right)-m_{11}\left(t\right),\\ h_{12}\left(t\right) & =s_{11}\left(t\right)-m_{12}\left(t\right),\\ \vdots \\ h_{1n}\left(t\right) & =s_{1(n-1)}\left(t\right)-m_{1n}\left(t\right). \end{array}\right. \end{array}$$

where

(6) $$\begin{array}{c} \left\{\begin{array}{cc} s_{11}\left(t\right) & =\frac{s_{11}\left(t\right)}{a_{11}\left(t\right)},\\ s_{12}\left(t\right) & =\frac{s_{12}\left(t\right)}{a_{12}\left(t\right)},\\ \vdots \\ s_{1n}\left(t\right) & =\frac{s_{1n}\left(t\right)}{a_{1n}\left(t\right)}. \end{array}\right. \end{array}$$

The corresponding envelope signal is as follows:

(7) $$a_{1}\left(t\right)=a_{11}\left(t\right)a_{12}\left(t\right)\cdots a_{1n}\left(t\right)=\prod_{q=1}^{n}a_{1q}\left(t\right),$$

and the objective is

(8) $$\lim_{n\to \infty }a_{1n}\left(t\right)=1.$$

Step 4 The first product function $PF_{1}$ of the original signal is derived through the multiplication of the envelope signal $a_{1}\left(t\right)$ with the pure frequency-modulated signal $s_{1n}\left(t\right)$.

(9) $$PF_{1}\left(t\right)=a_{1}\left(t\right)s_{1n}\left(t\right),$$

In $PF_{1}$, the highest-frequency constituent of the original signal is encapsulated within a single-component AM-FM signal, where its instantaneous amplitude faithfully aligns with the envelope signal $a_{1}\left(t\right)$. The instantaneous frequency, dictated by the pure frequency-modulated signal $s_{1n}\left(t\right)$, can be mathematically expressed as

(10) $$f_{1}\left(t\right)=\frac{1}{2\pi }\frac{d[\arccos\left(s_{1n}\left(t\right)\right]}{dt}.$$

From the original data x(t), subtracting the product function yields a new function $u_{1}\left(t\right)$, representing a smoothed version of the original data. The entire process is repeated *n* times until $u_{n}\left(t\right)$ becomes a constant or no longer contains oscillations.

(11) $$\begin{array}{c} \left\{\begin{array}{cc} u_{1}\left(t\right) & =x\left(t\right)-PF_{1}\left(t\right),\\ u_{2}\left(t\right) & =u_{1}\left(t\right)-PF_{2}\left(t\right),\\ \vdots \\ u_{k}\left(t\right) & =u_{k-1}\left(t\right)-PF_{k}\left(t\right). \end{array}\right. \end{array}$$

Step 5 Finally, the original signal x(t) is decomposed into an nth-order product function and a monotonic function $u_{k}$.

(12) $$x\left(t\right)=\sum_{p=1}^{n}+u_{n}.$$

### 3.2. Robust Local Mean Decomposition

RLMD addresses some of the shortcomings of LMD. LMD has numerous advantages compared to previous algorithms. Particularly, it can avoid the presence of negative frequencies in decomposed subsequences [31]. However, end effects and mode mixing can interfere with the effectiveness of LMD-decomposition. Typically, to overcome these two limitations, it is necessary to predetermine appropriate parameters related to boundary conditions, envelope estimation, and filtering stop criteria [23]. This process often consumes a significant amount of time and, at times, faces limitations in determining parameters due to experimental conditions. In light of this, RLMD is proposed, optimizing specifically as follows:

(1) Determine boundary conditions: The mirrored extension algorithm is first proposed to determine symmetric points on both sides of the signal.

(2) Envelope signal estimation

(13) $$\lambda^{*}=\mathrm{odd}\left(\mu_{s}+3\times \delta_{s}\right),$$

(14) $$\mu_{s}=\sum_{i=1}^{K-1}s\left(i\right)\cdot f\left(i\right),\;\delta_{s}=\sqrt{\sum_{i=1}^{K-1}{\left[s\left(i\right)-\mu_{s}\right]}^{2}\cdot f\left(i\right)}.$$

s(·) is the step length, f(·) is the corresponding probability density function, odd(·) is designed to round up to the nearest odd integer, and $\mu_{s}\;and\;\;\delta_{s}$ are the mean and standard deviation, respectively.

(3) Sifting criterion minimize F

(15) F=RMS[z(t)]+EK[z(t)],

where z(t)=a(t)−1 is the zero-baseline envelope. a(t) is the original signal.

(16) $$\mathrm{RMS}\left[z\left(t\right)\right]=\sqrt{\frac{1}{n}\sum_{t=1}^{n}{\left[z\left(t\right)\right]}^{2}},$$

(17) $$\mathrm{EK}\left[z\left(t\right)\right]=\frac{\frac{1}{n}\sum_{t=1}^{n}{\left[z\left(t\right)-\bar{z}\right]}^{4}}{{\left(\frac{1}{n}\sum_{t=1}^{n}{\left[z\left(t\right)\right]}^{2}\right)}^{2}},\;\bar{z}=\frac{1}{n}\sum z\left(t\right).$$

More details of RLMD can be found in [23].

### 3.3. Sample Entropy

SE [22] is an algorithm for assessing the complexity of time series. By evaluating the likelihood of generating different patterns within a sequence, SE measures the complexity of the sequential signal. One of the key reasons for the widespread use of SE is its insensitivity to the length of the sequence; the accuracy of sample entropy is not affected by the length of the input sequential signal. Moreover, SE exhibits a high level of detection accuracy. Even with relatively short data, SE can provide stable estimates and demonstrate excellent consistency. In order to achieve a balance between denoising effectiveness and signal fidelity, this study employed a combination of SE and RLMD. The SE values are used to categorize the PF components obtained after the RLMD decomposition based on their SE values.

The algorithmic description of SE is as follows:

(1) For a time series {x(n)∣n=1,2,…,N}, re-establish a new sequence {X(n)}, where X(k)=(x(k),x(k+1),…,x(k+m−1)) is an *m*-dimensional vector, and k+m−1≤N. Utilize the newly created sequence {X(n)} for subsequent operations.

(2) Define the distance between $X(n_{1})$ and $X(n_{2})$ as follows:

(18) $$d_{n_{1},n_{2}}=d\left[X\left(n_{1}\right),X\left(n_{2}\right)\right]=\max_{i=0,1,...,m-1}\left|x\left(n_{1}+i\right)-x\left(n_{2}+i\right)\right|.$$

(3) For a fixed $n_{1}$ and m, find the set of all $n_{2}$ that satisfies the condition $\left\{n_{2}|d_{n_{1},n_{2}}<r,n_{2}\leq N-m\right\}$ and denote the size of this set as $B_{n_{1}}$. And, denote $B_{n_{1}}^{m}\left(r\right)$ as

(19) $$B_{n_{1}}^{m}\left(r\right)=\frac{1}{N-m-1}B_{i}.$$

(4) Denote

(20) $$B^{m}\left(r\right)=\frac{1}{N-m}\sum_{k=1}^{N-m}B_{k}^{m}\left(r\right).$$

(5) Increase the dimension of $X_{m}\left(n_{1}\right)$ by one, i.e., transform it into $X_{m+1}\left(n_{1}\right)$. And $A_{i}=\left|\left\{n_{2}|d\left[X_{m+1}\left(n_{1}\right),X_{m+1}\left(n_{2}\right)\right]<r,n_{2}\leq N-m\right\}\right|$, We define $A_{n_{1}}^{m+1}\left(r\right)$ as

(21) $$A_{n_{1}}^{m+1}\left(r\right)=\frac{1}{N-m-1}A_{i}.$$

(6) Define $A^{m}\left(r\right)$ as

(22) $$A^{m}\left(r\right)=\frac{1}{N-m}\sum_{k=1}^{N-m}A_{i}^{m}\left(r\right).$$

In this way, $B^{m}\left(r\right)$ can be interpreted as the probability of finding m points when two sequences are similar within a limit of *r*, while $A^{m+1}\left(r\right)$ represents the probability of finding *m* + 1 points. The definition of sample entropy is then written as

(23) $$\mathrm{SE}\left(m,r\right)=\lim_{N\to \infty }-\ln\frac{A^{m}\left(r\right)}{B^{m}\left(r\right)},$$

when *N* is limited, we have

(24) $$\mathrm{SE}\left(m,r,N\right)=-\ln\frac{A^{m}\left(r\right)}{B^{m}\left(r\right)}.$$

The larger the numerical value of sample entropy, the more regularity implied in the sequence. Conversely, higher values indicate greater irregularity and complexity in the sequence. In general, the sample entropy of pure noise signals is high. Currently, sample entropy is widely utilized in areas such as diagnosing pathological conditions and fault detection.

### 3.4. Time-Frequency Peak Filtering

Mesbah et al. [32] introduced a filtering technique named TFPF. This technique has the capability to extract useful signals in noisy environments and is currently widely applied in various engineering fields. The principle of the TFPF algorithm relies mainly on the Wigner–Ville distribution and the theory of instantaneous frequency estimation. It involves encoding noisy signals, transforming them into analytic signals of instantaneous frequency, and then estimating their instantaneous frequency to obtain effective values.

Let the noisy data to be processed be represented as follows:

(25) s(t)=x(t)+n(t).

where *x*(*t*) is the effective signal; *n*(*t*) is additive random noise. The purpose of filtering is to recover the effective signal *x*(*t*) from the noisy data *s*(*t*).

TFPF can effectively recover the effective signal without making assumptions. The specific steps are as follows

(1) Perform scale transformation on the original signal. To avoid the discontinuity of instantaneous frequency at the frequency boundaries in the time-frequency plane and ensure effectiveness, an appropriate frequency range should be selected for scaled transformation. The scale transformation formula is

(26) $$x_{c}\left(m\right)=\left(a-b\right)\frac{x(m)-min[x(n)]}{max[x(m)]-min[x(m)]}+b.$$

where $x_{c}\left(m\right)$ is called the transformed signal; x(m) is the sampled signal, m=0,1,2,….

At this point, $a=\max[x_{c}\left(m\right)]\leq 0.5$, $b=\min[x_{c}\left(m\right)]\geq 0$. Changing *a* and *b* can adjust the frequency limits of the encoded signal.

(2) Modulate the scaled transformed signal *x*(*t*) in frequency to obtain a unit-amplitude analytic signal

(27) $$z_{x}\left(t\right)=e^{j2\pi \mu \int_{0}^{t}x\left(\lambda \right)d\lambda }.$$

where μ is scaling factor.

(3) Take the pseudo-Wigner–Ville distribution peaks of the analytic signal z(t) and perform instantaneous frequency estimation on the analytic signal, yielding an estimate for the effective signal $\hat{s}\left(t\right)$

(28) $$\hat{s}\left(t\right)=\frac{\arg\max[W_{z}\left(t,f\right)]}{\mu }.$$

(4) Recover signal $\hat{s}\left(m\right)$

(29) $$\hat{s}\left(m\right)=\frac{\left[{\hat{s}}_{c}\left(m\right)-b\right]\left\{max\left[x\left(m\right)\right]-min\left[x\left(m\right)\right]\right\}}{a-b}+min\left[x\left(m\right)\right].$$

The most crucial parameter in the TFPF algorithm is the window length. When the window length is set to be large, noise elimination is more thorough, but it comes at the cost of greater loss of effective signals. Conversely, with a smaller window length, the effectiveness in noise elimination is weakened. Therefore, choosing an appropriate window length is critical. In this paper, we employ a combination of the RLMD decomposition algorithm and sample entropy. Different window lengths are selected for various segments to make the filtering more targeted.

### 3.5. Temperature Compensation Model

**Figure 6:**
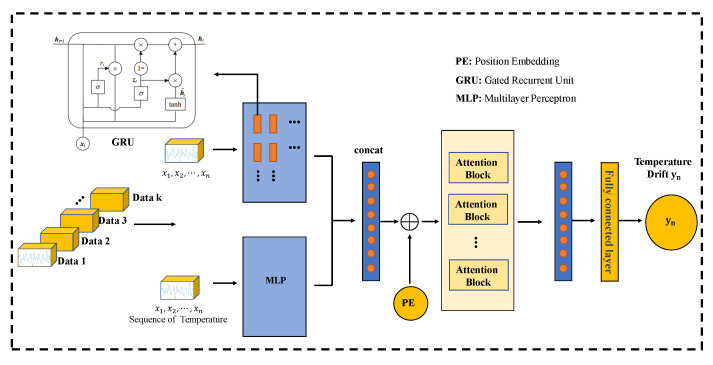
Architecture of the proposed GMAN model for temperature drift prediction.

This section details a temperature compensation model GMAN based on GRU, MLP, and attention. The architecture of the proposed GMAN model is illustrated in Figure 6. The GMAN model possesses several key features that contribute to its superior performance, particularly in accurately predicting sharp changes in temperature input and achieving robust compensation results for accelerometer output signals.

Firstly, our GMAN model adopts a three-stage structure. In the first stage, data are fed into a basic GRU module (two layers) and a simple MLP network, with their outputs concatenated. This structure is advanced in handling abrupt temperature changes. Simultaneously, the data are also fed into an MLP network in parallel to ensure the model has a sufficiently large function space while retaining the original input information for the next stage.

Secondly, the attention module in the second stage of the GMAN model plays a crucial role in handling cases where temperature sequences change gradually. Specifically designed to process sequence signals, the attention mechanism allows the model to dynamically focus on relevant parts of the input sequence, effectively capturing the evolving patterns in temperature data. By attending to important temporal information, the model can better understand the gradual changes in temperature over time and incorporate this knowledge into its predictions. Furthermore, the final fully connected layer in the GMAN model serves to increase the model’s function space while reducing multidimensional signals to single-dimensional ones. This transformation helps distill the essential features extracted from the temperature and accelerometer data, facilitating the model’s decision-making process and enhancing its predictive performance.

Overall, the three-stage structure of the GMAN model enables it to fully utilize the characteristics of different stages, thus better handling complex temperature variations. By incorporating attention mechanisms and fully connected layers, the model can effectively capture the nuanced relationships between temperature input and accelerometer output, leading to more accurate and robust predictions.

In addition to its structural advantages, the GMAN model exhibits strong adaptability and generalization ability. It can effectively learn and capture the complex relationships between temperature input and accelerometer output, without being limited by specific datasets or environmental conditions. This flexibility allows the model to perform exceptionally well in various real-world applications and achieve robust compensation for accelerometer output signals under different circumstances.

Through our GMAN model, we can obtain the prediction of temperature drift for the current moment.

(30) $$\mathrm{Input}=[x_{1},x_{2},\cdots ,x_{n}],$$

where *n* is 100.

(31) $$\mathrm{Output}=\mathrm{GMAN}(\mathrm{Input}),\;\mathrm{Output}\;\in R^{1}\;.$$

The training data in this study comprised 500,000 accelerometer outputs under different temperature conditions, ensuring high accuracy in temperature prediction by the model.

#### 3.5.1. Gated Recurrent Unit Block

GRU is a specialized type of recurrent neural network (RNN), proposed alongside long short-term memory (LSTM) [33,34], to address the gradient vanishing problem inherent in traditional RNNs. GRU provides a more comprehensive consideration of long- and short-term dependencies in time series data. Compared to LSTM, GRU exhibits faster convergence while maintaining comparable accuracy. As a result, it has found applications in the field of short-term load forecasting.

GRU consists of an update gate and a reset gate. Compared to LSTM, GRU has fewer parameters with three gate structures, resulting in faster convergence. The update gate controls the extent to which information from the previous time step is retained in the current state, where a larger value indicates more retention of the previous state information. The reset gate controls the degree to which the current state is combined with previous information, with a smaller value implying more ignored information. In the diagram, the direction of the arrows indicates the flow of data, σ represents the sigmoid activation function, tanh is the tanh activation function. It consists of a hidden state $h_{t}$, an update gate $z_{t}$, a reset gate $r_{t}$, and a candidate hidden state ${\tilde{h}}_{t}$. The update gate, reset gate, candidate hidden state, and final hidden state are determined by

(32) $$z_{t}=\sigma \left(W_{z}\cdot \left[h_{t-1},x_{t}\right]+b_{z}\right),$$

(33) $$r_{t}=\sigma \left(W_{r}\cdot \left[h_{t-1},x_{t}\right]+b_{r}\right),$$

(34) $${\tilde{h}}_{t}=\tanh\left(W_{h}\cdot \left[r_{t}⊙h_{t-1},x_{t}\right]+b_{h}\right),$$

(35) $$h_{t}=\left(1-z_{t}\right)⊙h_{t-1}+z_{t}⊙{\tilde{h}}_{t}.$$

This architecture enables GRU to effectively manage and leverage contextual information across various time steps.

#### 3.5.2. Attention Mechanism

The attention mechanism, inspired by the human brain’s selective focus, allocates resources by concentrating on specific regions at a given moment. This emulation of cognitive attention aims to enhance model accuracy by cleverly adjusting the attention to information, ignoring irrelevant details, and amplifying the necessary signals. Utilizing a probabilistic allocation approach, attention assigns sufficient focus to key information, highlighting the impact of critical details and ultimately improving model accuracy. Here, we adopted self-attention to dynamically assign importance to different elements within a sequence.

For regular dot-product attention, matrices $Q,K,V\in R^{L\times d}$ represent intermediate representations of the input, with rows serving as queries, keys, and values. Bidirectional [35] dot-product attention, represented by $A\in R^{L\times L}$, has the form

(36) $$\mathrm{Attention}\left(Q,K,V\right)=D^{-1}AV,\;A=\exp\left(\frac{QK^{T}}{\sqrt{d}}\right),\;D=\mathrm{diag}\left(A1_{L}\right).$$

Here, exp() is applied elementwise, $1_{L}$ is the all-ones vector of length *L*, and diag() is a diagonal matrix. The time and space complexities of computing this attention are $O(L^{2}d)$ and $O(L^{2}+Ld)$, respectively. Bidirectional attention is used in Seq2Seq architectures.

Unidirectional dot-product attention, represented by $\hat{\mathrm{Attention}}\left(Q,K,V\right)$, has the form

(37) $$\hat{\mathrm{Attention}}\left(Q,K,V\right)={\hat{D}}^{-1}\hat{A}V,\;\hat{A}=\mathrm{tril}\left(A\right),\;\hat{D}=\mathrm{diag}\left(\hat{A}1_{L}\right).$$

Here, tril() returns the lower-triangular part of the matrix, including the diagonal. Unidirectional attention is used for autoregressive generative modeling, such as self-attention in generative transformers and the decoder part of Seq2Seq transformers.

In this paper, for a given input sequence $X=\{x_{1},x_{2},\cdots ,x_{L}\}$, where *L* is 100, let us delve into the detailed computational steps of the attention mechanism

Compute query, key, and value vectors

(38) $$Q=W_{q}X,\;K=W_{k}X,\;V_{i}=W_{v}X.$$

Compute attention scores

(39) $$\mathrm{Attention}(Q,K,V)=\mathrm{softmax}(\frac{QK^{T}}{\sqrt{d}})V.$$

In addition, due to the abandonment of recursive structures and convolution in the computation of transformers, it is unable to simulate the positional information of sequential inputs. Therefore, positional encoding is artificially added. Each unit in the sequence is assigned a positional index, and each index corresponds to a vector. By combining the positional vector with the input vector, positional encoding introduces positional information to each input.

(40) $$\begin{array}{c} \left\{\begin{array}{cc} \mathrm{PE}(\mathrm{pos},2i) & =\sin(\mathrm{pos}/10,000^{2i/d}),\\ \mathrm{PE}(\mathrm{pos},2i+1) & =\cos(\mathrm{pos}/10,000^{2i/d}). \end{array}\right. \end{array}$$

Following this, the output generated by the attention mechanism module is fed into a fully connected layer, resulting in the final temperature drift prediction for the nth time point.

### 3.6. The Structure of the Temperature Compensation Model

**Figure 7:**
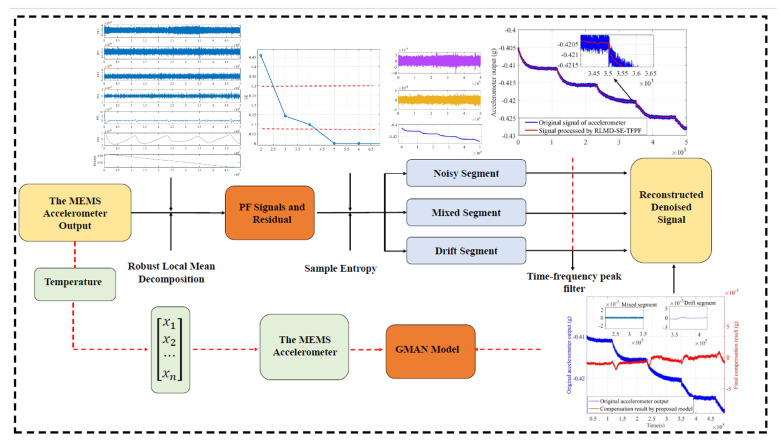
Flow chart of RLMD-SE-GMAN parallel model.

Figure 7 is the parallel processing model of RLMD-SE-GRU-Attention proposed in this paper. The algorithm is as follows:

a. We employ RLMD to decompose the accelerometer signal into multiple PF functions and residuals. Subsequently, we use SE to analyze each component based on the continuity’s autocorrelation and complexity, dividing the signal into three segments: noise, mixed, and drift. The noise segment exhibits a considerable amount of chaotic behavior, resembling white noise. Meanwhile, the mixed segment contains both noise and drift trends. Therefore, we apply a larger filtering window with the TFPF algorithm to process the noisy segment and a relatively smaller window for the drift segment, utilizing the TFPF algorithm for filtering.

b. To enhance algorithm efficiency and accuracy, we employ a GMAN model for temperature compensation, considering the strong lag of temperature. The model uses past moments’ temperature time series as input, providing a more accurate prediction of the current moment’s temperature drift and aligning better with its influence on accelerometer output. After training, a temperature drift prediction model for the current moment is obtained.

c. By reconstructing the processed accelerometer output, we obtain the final compensation signal. This result reflects the outcome of temperature error processing, eliminating high-frequency noise and low-frequency drift.

However, given the application of the hybrid parallel algorithm based on RLMD-SE-TFPF and GMAN for temperature compensation and denoising in single-chip three-axis capacitive MEMS accelerometers, there are several challenges and limitations associated with implementing parallel processing methods. Firstly, this method compensates for temperature and denoises accelerometer outputs over a period of time, but it exhibits significant limitations when processing point-to-point output signals. Specifically, the algorithm’s denoising capability is severely affected when there is only one temperature input and its corresponding accelerometer output. Additionally, for predicting temperature drift based on n identical temperature sequences after GMAN model input completion, GMAN no longer provides a distinct advantage over other point-to-point prediction algorithms. Secondly, the proposed hybrid parallel algorithm requires a certain level of computational power, which may not be conducive to deployment on the edge networks of MEMS accelerometers. This could limit the algorithm’s application in resource-constrained devices or edge computing environments where sufficient computational resources may not be available to support parallel processing.

Therefore, while the hybrid parallel algorithm has potential advantages in temperature compensation and denoising, these challenges and limitations need to be carefully considered in practical applications to ensure the effectiveness and reliability of the algorithm.

## 4. Expriments and Analysis

### 4.1. Temperature Experiment

**Figure 8:**
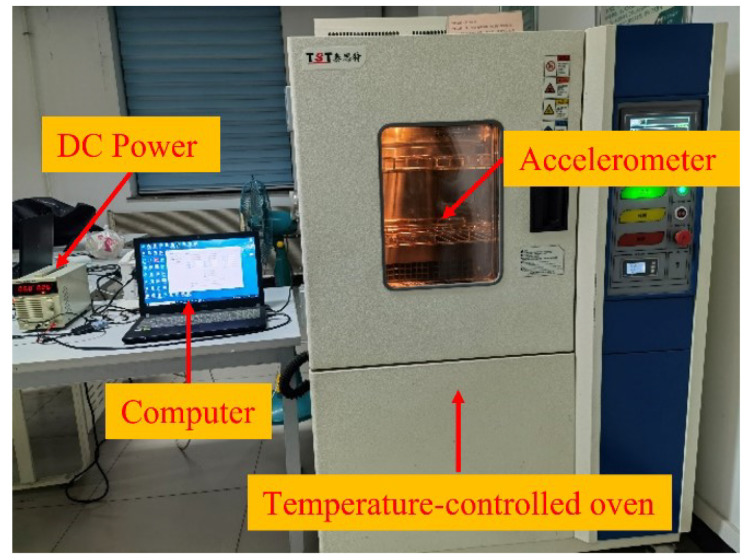
Temperature measuring equipment.

**Figure 9:**
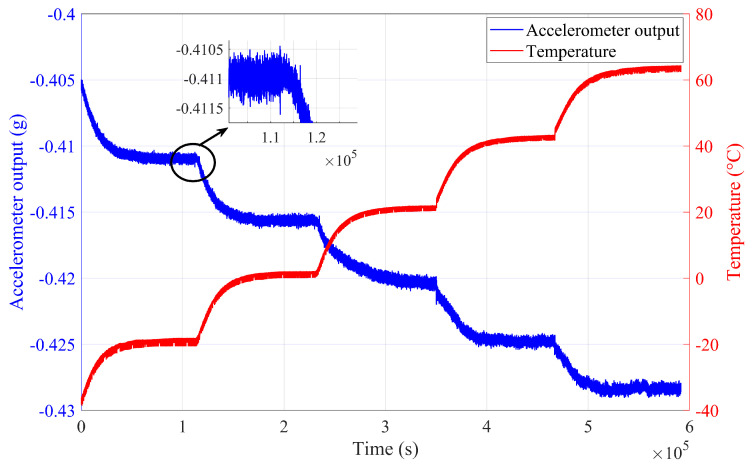
Temperature experiment results in atemperature-controlled oven.

We conducted temperature drift experiments to validate the RLMD-SE-GRU-Attention parallel model proposed above. Through temperature experiments, we tested the temperature characteristics of a single-chip capacitive MEMS accelerometer. In the temperature experiment, a temperature-controlled oven ranging from −40 °C to +150 °C (see Figure 8) was utilized. Data acquisition software collected the accelerometer’s output, powered by a Gwinstek GPS-4303C DC power supply sourced from Shenzhen Gwinstek Instrument Co., Ltd., Shenzhen, China. Real-time temperature within the metal casing was synchronized with the accelerometer’s output via a temperature sensor. The room temperature indoors was maintained at 25 °C. Initially, the accelerometer was secured on a stationary plane to avoid motion interference. Subsequently, the output lines were connected to the Gwinstek DC power supply and a laptop. The temperature of the oven was then set in the range of −30 °C to +60 °C. Finally, the power was switched on, and the original output signal of the accelerometer was recorded. The data acquisition process was continuous, and the results of the temperature experiment are depicted in Figure 9. As observed in Figure 9, the change in the accelerometer output became more pronounced with temperature variations. This phenomenon indicated that temperature significantly influenced the accuracy of the accelerometer output.

### 4.2. Results Analysis

**Figure 10:**
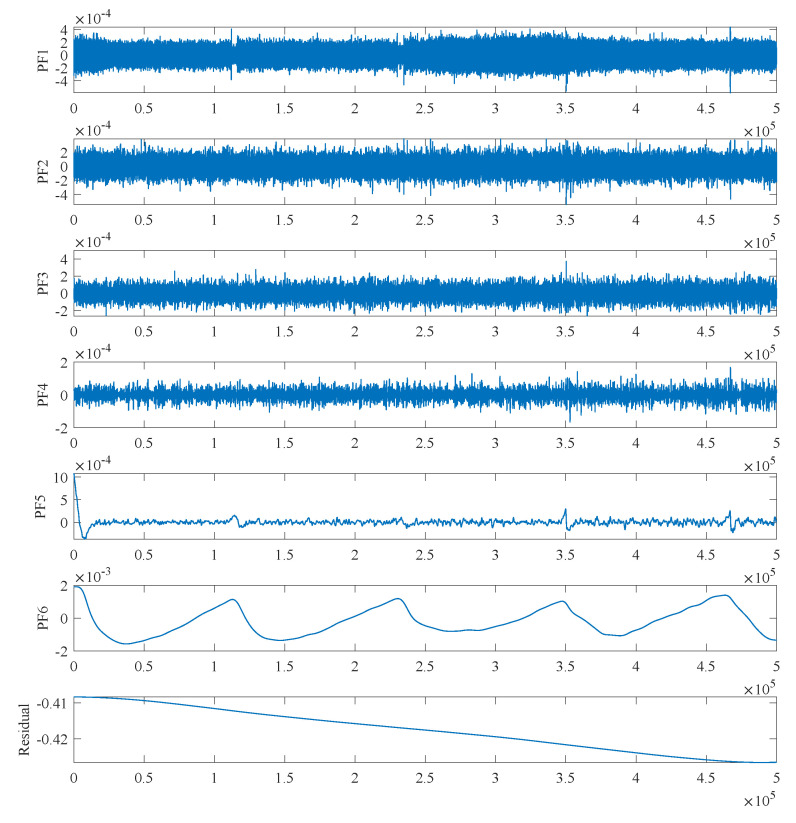
The decomposition results of accelerometer signal by RLMD.

From Figure 9, it can be observed that the output of the accelerometer undergoes significant changes with temperature variations, accompanied by a considerable amount of noise. Following the steps of the RLMD-SE and TFPF denoising algorithm, the output signal is first subjected to RLMD decomposition. Figure 10 illustrates the RLMD decomposition graph of the accelerometer output. After decomposition, components in multiple frequency bands are generated.

When utilizing sample entropy (SE) to analyze the segments of accelerometer output after being decomposed by the RLMD algorithm, a crucial aspect is how to determine the SE boundary.

Firstly, the fundamental concept of the RLMD method involves decomposing the signal into local mean components representing the smoothed portions of the signal within different frequency ranges. Specifically, RLMD decomposition involves convolving the signal with moving average filters of varying scales to obtain different frequency components. Each local mean component is derived from the difference between the original signal and its corresponding scale’s smoothed version. By iteratively applying this process, the signal is gradually decomposed until a certain stopping criterion is met or a specified decomposition level is reached. One of the primary advantages of RLMD is its ability to adaptively capture different frequency components within the signal without requiring prior knowledge or manual selection of filters. Consequently, the signals produced by RLMD better reflect the local characteristics of the original signal and provide a multiscale decomposition of the signal spectrum. Due to the reduced overlap of segments in the signal produced after RLMD decomposition, there is less demand for strict SE boundary determination.

**Figure 11:**
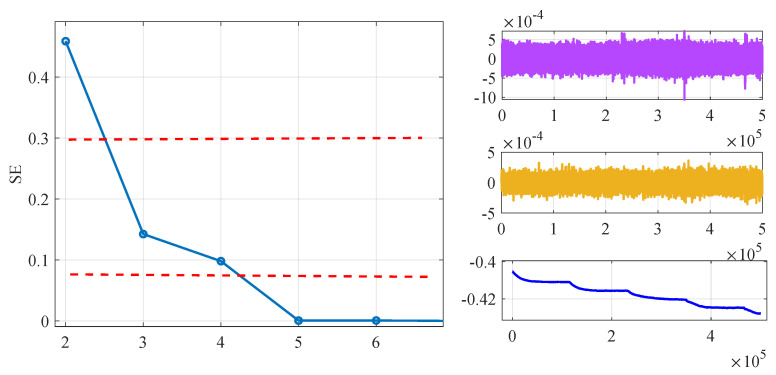
Classification of PF components according to SE values.

On the other hand, among the different components generated by RLMD decomposition of accelerometer signals, those with deeper iteration levels and residual components account for a larger proportion. Conversely, components with lower iteration levels exhibit minimal numerical values and chaotic effects, making them more conducive to the classification of noise, mixed, and drift segments. Figure 10 and Figure 11 illustrate this point. Therefore, the RLMD algorithm not only reduces the overlap of segments in the decomposed signal but also facilitates the partitioning of segments into noise, mixed, and drift categories, contributing to more effective determination of the SE boundary.

**Figure 12:**
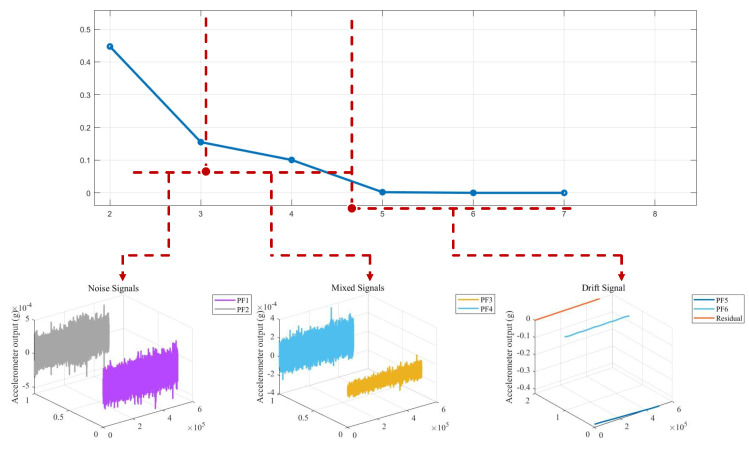
Calculation results according to SE and noise signals, mixed signals, drift signals.

Subsequently, the SE algorithm was employed to calculate the SE values of the six PF components and the Res component. These seven components were then classified into three segments based on their SE values. As depicted in Figure 11 and Figure 12, SE values ranged from 0 to 0.5. Signals with SE values greater than 0.3, including PF1 and PF2, had a value range between $-5.1\times 10^{-4}$ and $5.1\times 10^{-4}$, and their output did not exhibit drift with temperature changes. Therefore, this segment was identified as the noise segment, containing a significant amount of noise. Signals composed of PF3 and PF4 with SE values between 0.075 and 0.3 also exhibited a noise distribution, but this segment showed some drift with temperature changes and was identified as a mixed segment. The signals composed of the remaining PF components and residual component with SE values less than 0.075, as seen in Figure 11 and Figure 12, had curves that were generally smooth and exhibit noticeable drift with temperature changes, belonging to the drift segment.

**Figure 13:**
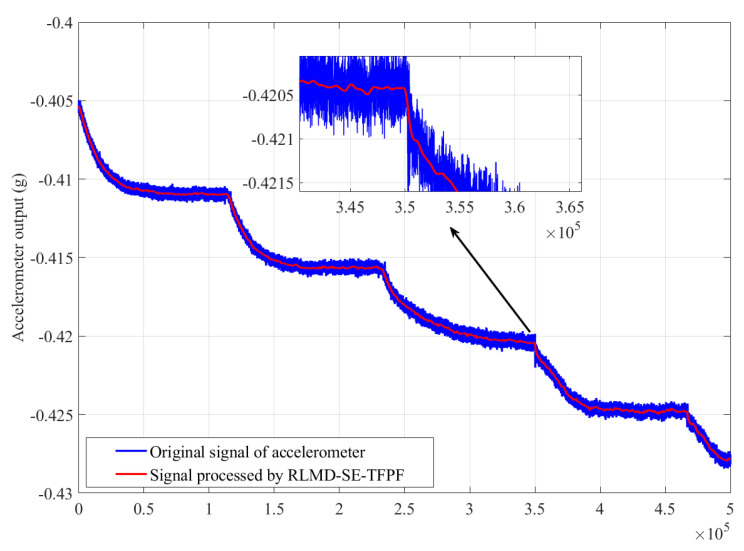
Signal processed by RLMD-SE-TFPF and original accelerometer output signal.

Figure 13 illustrates a comparison between the signal processed by RLMD-SE-TFPF and the original accelerometer signal. Reconstruction was performed on the output of the noise segments and mixed segments after TFPF to obtain the denoised signal. Comparing the denoised signal with the original accelerometer output signal, the TFPF algorithm after RLMD-SE processing effectively removes the influence of noise. The RLMD-SE-TFPF methods after processing effectively eliminate the influence of noise.

**Figure 14:**
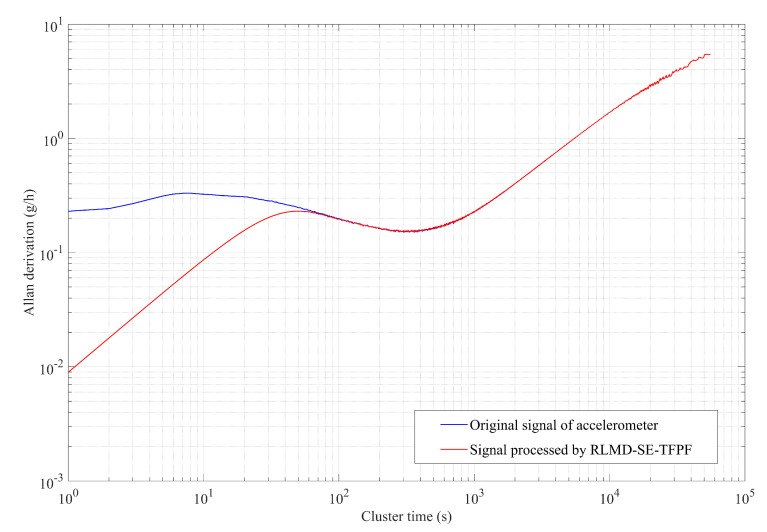
The Allan derivation of signal processed by RLMD-SE-TFPF and original accelerometer output signal.

Allan variance [36] is a time-domain analysis technique commonly used for analyzing random errors in data collected by accelerometers under static conditions. Here, the Allan variance curve was used to analyze the denoising effects of the RLMD-SE-TFPF methods, as shown in Figure 14. The zero-deviation stability of the original accelerometer output signal and the signal processed by the RLMD-SE and TFPF algorithms were 20.2497 g/h and 20.2473 g/h, showing a slight decrease. The acceleration random walk (representing noise characteristics) was reduced by 96.11%, with values of 0.23032 g/h$/\sqrt{\mathrm{Hz}}$ for the original signal and 0.00895893 g/h$/\sqrt{\mathrm{Hz}}$ for the processed signal. This quantitative analysis confirmed the effectiveness of our decomposition denoising algorithm.

**Figure 15:**
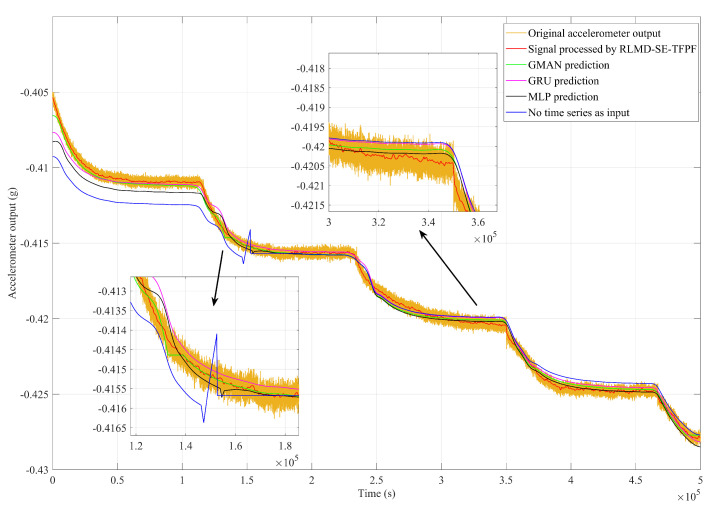
The results of the temperature compensation based on four methods.

To validate the superiority of our GMAN algorithm, this study conducted multiple sets of ablation experiments. In Figure 15, we demonstrate the predicted outputs of the GMAN model, GRU model, and simple MLP model when time series data were used as input. Upon inspection of the results, it is evident that the GMAN model performs remarkably well in scenarios involving sudden changes and at the endpoints of the time series, outperforming both the GRU and MLP models. This improvement in accuracy validates the effectiveness of integrating GRU and attention mechanisms within our model architecture.

Furthermore, when the temperature deviates from sharp points of change, the input of the GRU model tends to be higher than the actual value, potentially due to the inherent forgetting properties of the GRU model. Additionally, observing the temperature drift curve predicted by the MLP model reveals significant oscillations compared to the actual values, possibly resulting from overfitting during training due to the nature of MLP model as a network architecture. Our proposed GMAN model mitigates the effects to some extent the shortcomings of both the GRU and MLP models and incorporates an attention module, yielding superior performance compared to the individual GRU and MLP models.

Moreover, supplementary experiments were conducted by training the network with a single temperature value as input. The experiments showed a noticeable decrease in prediction accuracy, especially during temperature transitions. This outcome underscores the importance of utilizing time series data as input, which allows our model to capture the inherent temporal dependencies and fluctuations in the data, thereby enhancing predictive performance. This experiment confirmed the superiority of using time series as input.

Additionally, we adopted a rigorous experimental design to ensure fair comparisons between the models. This included controlling variables such as the composition of the dataset, training parameters, fixed random seeds, and evaluation metrics to minimize the influence of external factors on the results.

Furthermore, extensive sensitivity analyses were conducted to identify and mitigate any potential biases or confounding factors. This involved systematically varying experimental conditions and assessing the robustness of the models’ performance across different scenarios. By thoroughly examining the sensitivity of the models to various factors, any potential sources of bias in the analysis could be identified and appropriately addressed.

Additionally, in conducting Allan variance calculations, we controlled for the selection of input from the same data segments and maintained consistent parameters. This helped ensure that any differences in prediction accuracy could confidently be attributed to inherent differences between the models under study. Overall, through carefully designed experiments, sensitivity analyses, and statistical controls, we were able to address potential biases or confounding factors and provide robust comparisons of GMAN, GRU, and MLP models in terms of prediction accuracy.

**Figure 16:**
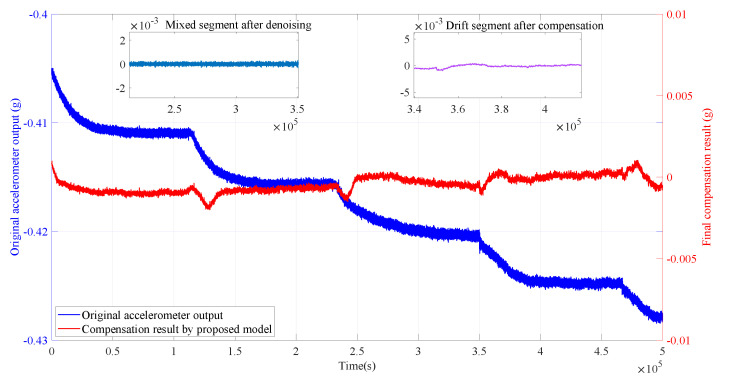
The final accelerometer output signal after denoising and compensation.

**Figure 17:**
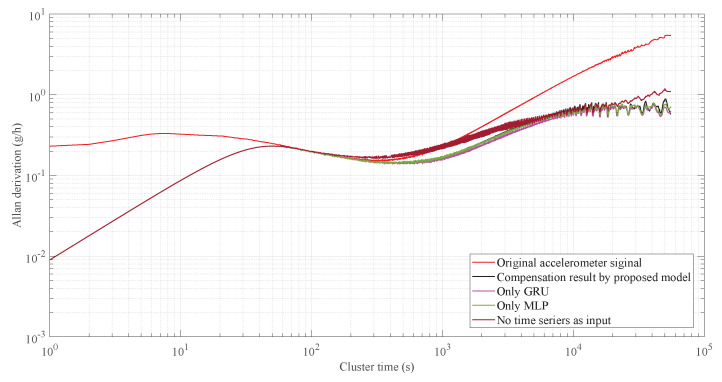
The Allan variance derivation of the compensation results of accelerometer based on four methods.

We wrote the program on Visual Studio Code and ran the GMAN model on an Nvidia RTX 4090. Based on our experimental findings, the GMAN model typically takes a few milliseconds to compute on a GPU. Conversely, the computation time of the RLMD-SE-TFPF methods depends heavily on the sequence length. For instance, on common CPUs, the execution time is around 10 ms for sequences with lengths not exceeding 100. For sequences of length 10,000, the execution time varies between 100 and 300 ms. However, when the sequence length exceeds 200,000, the computation time exceeds 3 min. Typically, RLMD-SE-TFPF processes sequences of around 100 in length. When combined with the inference time of the GMAN model, the runtime of this parallel temperature compensation algorithm generally remains below 10 ms, making it fully suitable for practical applications. Figure 16 showcases the final compensation results obtained through the GMAN temperature drift prediction model and the RLMD-SE-TFPF decomposition denoising algorithm. The results indicate that our proposed solution effectively suppressed the noise in the accelerometer output and compensated for temperature drift. Simultaneously, we quantitatively compared the temperature compensation results obtained using different temperature drift prediction models using Allan variance, as shown in Figure 17.

Our algorithm achieved a greater reduction in random walking compared to Zhu et al. [9] RBF NN + GA + KF method (96.11% reduction versus 95.53% reduction), as well as compared to Guo et al. [37] DLSTM, RNN, and ISSA method (without denoising decomposition algorithm) (96.11% reduction versus 57.40% reduction), and Gang et al. [38] GRU, RNN, short GRU, and optimized monarch butterfly method (96.11% reduction versus 92.15% reduction). This indicates that our method provides superior accuracy in reducing acceleration random walking.

Our proposed solution, compared to using only the GRU module, only using MLP as the temperature drift prediction model, and not using time series as the model input, achieves zero-deviation stability values of 1.76137 g/h, 1.34093 g/h, 1.58005 g/h, and 3.29027 g/h, respectively. The corresponding acceleration random walk values are 0.00895695 g/h$/\sqrt{\mathrm{Hz}}$, 0.00895677 g/h$/\sqrt{\mathrm{Hz}}$, 0.00895745, and 0.00896909 g/h$/\sqrt{\mathrm{Hz}}$. The quantitative results demonstrate that our proposed temperature compensation solution outperforms other models. Compared to the original accelerometer output with zero-deviation stability and acceleration random walk values of 20.2497 g/h$/\sqrt{\mathrm{Hz}}$ and 0.00895695 g/h$/\sqrt{\mathrm{Hz}}$, respectively, our proposed parallel temperature compensation model exhibits excellent performance.

## 5. Conclusions

This study investigated the structure of single-chip three-axis capacitive MEMS accelerometers and proposed a temperature compensation and denoising solution. A novel parallel processing method, named the hybrid parallel algorithm, was introduced based on RLMD-SE-TFPF and GMAN. Due to variations in accelerometer signals under different temperature conditions, where noise and temperature drift in the output signals differ, the RLMD algorithm is initially employed for signal decomposition. The SE values are calculated to assess the complexity of each component, categorizing the signal into noise segments, mixed segments, and temperature drift segments. This approach not only reduces the computational complexity of signal processing but also maximizes the retention of temperature drift components. Additionally, leveraging the characteristic of temperature hysteresis, this study used the temperature time series as input for the temperature drift prediction model, enhancing model accuracy. The architecture incorporates GRU and attention modules, aligning with the characteristics of sequence input. Temperature experiments, output image comparisons, and Allan variance analysis demonstrated the effectiveness of the proposed solution. In comparison to previous algorithms, it is innovative and superior.

## Figures and Tables

**Table 1:** The modified structural parameters of the sensor.

Parameter	Size	Parameter	Size
Device Thickness (μm)	60	Comb-Tooth Count (Single Side)	26
Mass Block Mass (μg)	560	X/Y Axis Cruciform Beam Size (μm)	800 × 15
Comb-Tooth Gap (μm)	3	Blocking Block Gap (μm)	2
Comb-Tooth Dimensions (μm)	300 × 8		

**Table 2:** The first six mode frequencies of the accelerometer.

Modal Order	1	2	3	4	5	6
**Frequency (Hz)**	8563.3	11,027	12,752	20,034	22,640	53,071

## Data Availability

The data presented in this study are available on request from the corresponding author.
